# Metabolic Evaluation of Synthetic Opioids on the Example of U-47700 with the Use of In Vitro and In Vivo Methods for Forensic Toxicology Application

**DOI:** 10.3390/toxics11030220

**Published:** 2023-02-25

**Authors:** Sebastian Rojek, Ewa Poljańska, Weronika Chaim, Martyna Maciów-Głąb, Beata Bystrowska

**Affiliations:** 1 Department of Forensic Medicine, Faculty of Medicine, Jagiellonian University Medical College, Grzegórzecka 16 Str., 31-531 Kraków, Poland; 2 Toxicology Research Group, Department of Toxicology, Jagiellonian University Medical College, Medyczna 9 Str., 30-688 Krakow, Poland; 3 Department of Biochemical Toxicology, Faculty of Pharmacy, Jagiellonian University Medical College, Medyczna 9 Str., 30-688 Krakow, Poland

**Keywords:** U-47700, in vivo and in vitro metabolic study, ADMET Predictor, intoxication, LC-MS/MS

## Abstract

Legal highs present a great threat to health, especially in groups of people experimenting with psychoactive substances. The lack of available knowledge on the biotransformation of these substances necessitates symptomatic treatment in the event of intoxication, which, unfortunately, may be ineffective. Opioids, including heroin analogues, such as U-47700, constitute a special group of designer drugs. In this study, a multi-directional approach to trace the biotransformation of U-47700 in living organisms was used. For this purpose, the in silico assessment (ADMET Predictor) was used first and then followed by an in vitro study using human liver microsomes and the S9 fraction. The biotransformation was then followed in an animal model (Wistar rats). Tissues such as blood, brain and liver were collected for analysis. The study was performed using liquid chromatography with tandem mass spectrometry (LC-MS/MS). The obtained results were compared to those obtained from the analysis of autopsy materials (cases analysed in the Toxicology Laboratory of the Department of Forensic Medicine, Jagiellonian University Medical College in Krakow).

## 1. Introduction

New psychoactive substances (NSPs) are one of the most serious toxicological threats today, affecting many areas of social life, including legislation, forensics and public health, in addition to being a significant clinical problem [1]. Some substances with a psychoactive effect (psychedelics, stimulants, etc.) are very often characterized by a strong effect on the nervous system and addictive properties and are subjectively perceived as positive (mood improvement, well-being, euphoria), thus motivating their repeated use. A particular problem is the introduction to the market of newly prepared NSPs that mimic classic narcotic drugs and psychoactive substances. These include various groups of compounds that, until recently, were not subject to the Act on Counteracting Drug Addiction, whose use carries serious health effects in the form of acute poisoning, including fatal poisoning [2,3,4].

The compound known as U-47700 was developed in 1970 by Upjohn Company as a potential opioid analgesic, but research on it was abandoned and it was never approved for medical use. The full name of U-47700 according to the IUPAC is 3,4-dichloro-*N*-[(1R, 2R)-2-(dimethylamino)-cyclohexyl]-*N*-methylbenzamide. U-47700 is an alkaline lipophilic compound, very soluble in an acidic environment, and its physicochemical parameters are poorly understood [5,6]. During inaccurate synthesis, impurities are formed, which give the final product a slightly pink colour, hence its being commonly known as “pinky”. U-47700 works similarly to classic opioids, as a strong, selective agonist of the opioid receptor mu. The analgesic effect is estimated to be approximately 7.5 times stronger than morphine and approximately 10 times weaker than fentanyl in animal model studies, and the value of ED50 was determined at 0.2 mg/kg in the mouse tail flick assay [7].

To date, no detailed pharmacokinetic studies have been performed on U-47700, and the only available data come from the analysis of autopsy material and laboratory experiments. Four metabolites were identified in in vitro studies using human liver microsomes: *N*-desmethyl-U-47700 (M1), *N*,*N*-didesmethyl-U-47700 (M2), hydroxy-*N*-desmethyl-U-47700 (M3), and hydroxy-*N*,*N*-didesmethyl-U-47700 (M4). When comparing the results obtained with urine samples from poisoning victims, all of the metabolites (M1–M4) were observed, although only M1 and M2 were quantified (due to the lack of commercial standards of hydroxylation products) [8]. Recently, an in vivo study was also attempted in rats, again demonstrating the presence of U-47700 and its metabolites M1 and M2. The rats were administered three doses (0.3, 1 and 3.0 mg/kg) of U-47700. The dose and time-dependent concentration of metabolites were as follows: U-47700 dominated in the initial phase of the experiment and then decreased, inversely proportional to the concentration of metabolites. Halfway through the experiment’s duration (t = 480 min), *N*-desmethyl derivatives dominated; thus, at the end of the provided time, the concentration of the *N*,*N*-didesmethyl derivative increased [6].

Opioids were responsible for up to 74% of the fatal poisonings caused by psychoactive substances in 2019, according to the European Monitoring Centre for Drugs and Drug Addiction data. While heroin remains the most widely taken illicit opioid in Europe, and the drug accounts for the majority of drug-related deaths, there is growing concern about the role that other synthetic opioids play in Europe’s drug problem. Although the limited available data indicate that there has been a decline in both fatal and non-fatal fentanyl overdoses in 2020, there are signs that other synthetic opioids (not-fentanyl derivatives) may be playing a more significant role in drug problems in some countries [9].

Observing research directions in NSP forensic toxicology, it covers metabolomic is-sues using in vivo and in vitro models for obtaining data on the safety and pharmacology of these substances, especially soon after their introduction to the drug market. An open question in the area of research concerns the possibility of transferring these to data on a human model and using them for interpretation in death cases for medical–forensic opinion [10]. Tests were also conducted which compared the autopsy blood samples of 12 victims of U-47700 poisoning in which the presence of M1 and M2 was confirmed [11].

On the basis of the above experiences and observations, it can be seen that the basic reactions occurring during metabolic transformations of U-47700 are demethylation and hydroxylation; however, it is not known yet which cytochrome isoforms are responsible for these transformations, and phase II metabolism has not yet been studied.

## 2. Materials and Methods

### 2.1. Biological and Nonbiological Materials

#### 2.1.1. Animal and Sample Collection

The experiments were carried out on Wistar rats from the animal housing facility of the Jagiellonian University Medical College in Krakow. A group of 16 male Wistar rats (350–440 g) were kept in a natural day–night cycle at 22 ± 2 °C with food and water available ad libitum. All of the procedures were carried out according to the NIH Guide for the Care and Use of Laboratory Animals and were approved by the Local Ethics Committee (permission number: 306/2019). The animals were divided into two groups (controls—*n* = 4 and tested groups: *n* = 12/6 animals per dose). In the tested groups rats, were randomly placed into a two-dose administration group: 1 and 5 mg/kg. The animals were given a *p.o.* compound; then, after the appropriate time (30 and 90 min), they were sacrificed. The blood, brain and liver material for the study was collected. Selected tissues were immediately frozen in ice and stored at −20 °C.

#### 2.1.2. Chemicals and Reagents

The standards of *N*-desmethyl-U-47700 (purity ≥ 98%), *N*,*N*-didesmethyl-U-47700 and U-47700-*D*_6_ (CRM) were obtained from Cayman Chemical (Ann Arbor, MI, USA), and U-47700 (CRP) was obtained from Chiron (Trondheim, Norway). Acetonitrile and chloroform were purchased from Merck (Darmstadt, Germany), while methanol and formic acid were purchased from POCh (Katowice, Poland). All standard stock solutions were pre-pared in methanol (concentration 1 mg/mL) and stored at −20 °C. Further dilutions were prepared using acetonitrile. All chemical solvents and standards were of analytical grade. The 100 mM phosphate buffer (pH 7.4), prepared with ddH2O and 20 mM NADPH (Merck), was solubilized in 100 mM phosphate buffer.

Drugs: U-47700 (c = 1 mg/mL) dissolved in water and given *p.o.* in two doses: 1 and 5 mg/kg.

The in vitro study consisted of an experiment conducted using a commercially available pool of human liver microsomes (HLM) and S9 (human) enzyme fraction (Gibco^TM^, Thermo Fisher Scientific Inc., NYSE: TMO, Waltham, MA, USA). The HLM was pooled from 50 different individual donors. All of the donors were equally represented for a truer population sample. They were fully characterized for major cytochrome P450 activities and select phase II enzymes. The total protein concentration was 20 mg/mL.

The pooled human liver S9 fractions had a total protein concentration of 20 mg/mL. HLM and S9 were stored in an ultracold freezer (−80 °C) before use and protected from light.

### 2.2. Methods

#### 2.2.1. In Silico

**Figure 1:**
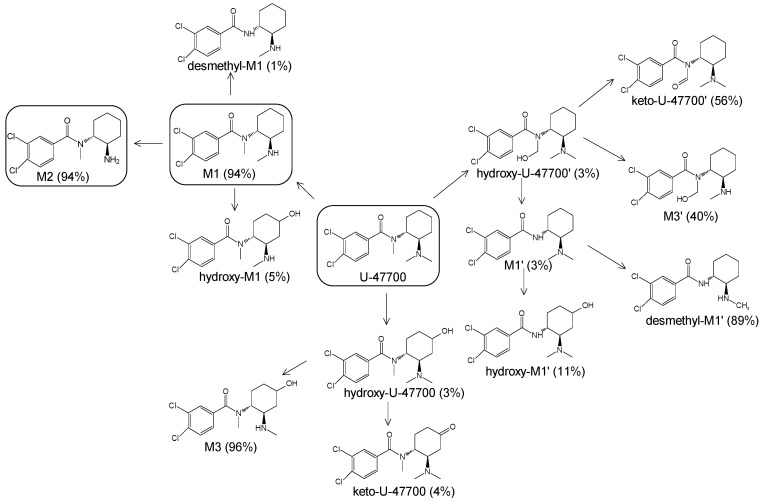
Suggested biotransformation pathways for U-47700 based on ADMET Predictor software.

The in silico evaluation included data analysis using the ADMET predictor (version 10.4.0.5; Simulations Plus, Inc.; Lancaster, CA, USA) software. Figure 1 shows the proposed U-47700 biotransformation pathways along with the percentage indicated by the program for a specific metabolite. An in silico study was performed as an introductory study for the actual U-47700 biotransformation study using microsomes, S9 fractions and hepatocytes. The ADMET Predictor program was used to determine the pathway of the first phase of the U-47700 metabolism.

Additionally, the in silico prediction software generated a list of accurate mass (expressed as *m*/*z* ratio in the inclusion list) for first-, second-, and third-generation predicted metabolites. This list was incorporated into the data-dependent MS2 (ddMS2) acquisition method operating in positive ESI, but identification of metabolites was not limited to this list. When a mass spectral peak on the inclusion list was detected, an MS/MS spectrum was automatically acquired.

#### 2.2.2. In Vitro Study

**Figure 2:**
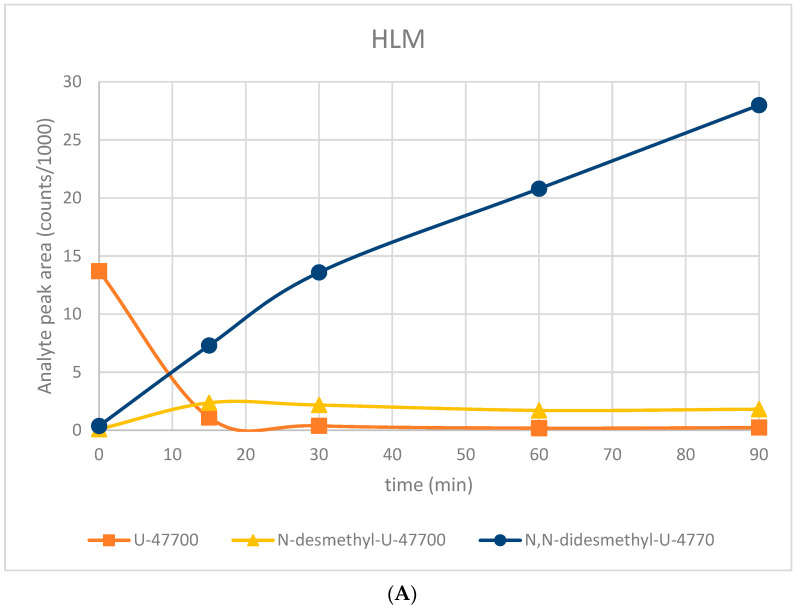

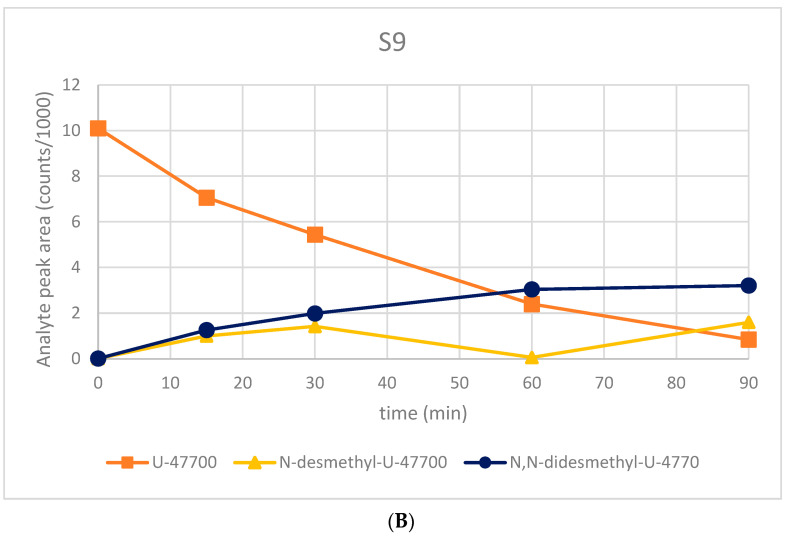
Graph showing changes in the amount of major metabolites of U-47700 over time after incubation with HLM (**A**) and S9 fraction (**B**).

The experiment consisted of incubating U-47700 (c = 150 µg/mL) at different time intervals (incubation with HLM and S9 fraction—time points: 0, 15, 30, 60, 90 min) according to the protocol recommended by Gibco. The proceedings included the addition of the following to the Eppendorf tubes: 183 µL of 100 mM phosphate buffer, 2 µL U-47700 solution and 5 µL of 20 mg/mL microsomes. The microsomes were pre-incubated with phosphate buffer, and U-47700 in a water bath for 5 min. The reactions were initiated with the addition of 10 µL 20 mM NADPH. Next, the samples were incubated for up to 90 min at 37 °C with gentle agitation. The reaction was terminated by the addition of 200 µL organic solvent—cold acetonitrile. The samples were vortexed and centrifuged at approximately 3000 rpm for five minutes. The supernatant was withdrawn from the protein pellet and analyzed using LC-MS/MS. The results are shown in Figure 2. The controls included a zero time point with U-47700 and the longest time point, without NADPH.

#### 2.2.3. In Vivo Study

The next stage of the study was an in vivo study in an animal model. Rats (males from the Wistar breed) were used for the study. U-47700 solution in physiological saline was administered *p.o.* through a gastric tube. Two doses (1 and 5 mg/kg b.w.) were used in the study. Animals from the experimental groups were divided into two sub-groups (according to the administered dose); three animals from each subgroup were then decapitated after both 30 min and after 90 min. The control animals received saline. After decapitation, blood and the liver and brain were collected from the animals. The blood was centrifuged and the serum was collected. All of the tissues were preserved until analysis (at a temperature of −20 °C).

### 2.3. U-47700 and Tissue Level Measurements by LC-ESI-MS/MS

#### 2.3.1. Extraction Procedure

The tissues were homogenized using a sonificator (UP50H, Dr. Hielscher GmbH, Teltow, Germany) in water in a proportionate 10 mg of wet tissue to 100 µL of solvent. Next, 500 μL of homogenate were mixed with 10 μL of internal standard (U-47700-*D*_6_, concentration 10 μg/mL), 500 μL of water, 250 μL of ammonia water (25%) and 3000 μL of ethyl acetate. The samples were vortexed for 30 s and centrifuged for 10 min at 2000 rpm. The organic phases were collected and dried under a stream of nitrogen at 40 °C. The residue was dissolved in 40 μL of acetonitrile and 10 μL of the reconstituted extract was injected into the LC-ESI-MS/MS system for quantitative analysis.

#### 2.3.2. LC-ESI-MS/MS Conditions

LC was performed using the LaChromElite system (Merck, Darmstadt, Germany), equipped with a degasser, binary pump, autosampler and thermostated column compartment (Merck, Darmstadt, Germany). Chromatographic separation was carried out with a Thermo Scientific BDS HYPERSIL C18 column (100 × 3 mm ID, 3 µm particle size; Thermo Fisher Scientific Inc., NYSE: TMO, Waltham, MA, USA) and precolumn (10 × 3 mm ID, 3 µm particle size). The advance column, along with its precolumn was set at 40 °C. The mobile phase was composed of 0.2% formic acid and 2 mM ammonium formate in water (B), and acetonitrile with 0.2% formic acid and 2 mM of ammonium formate (A). Chromatographic separation was achieved using the following gradient program: initially at 10% A for 1.5 min, before being increased linearly to 90% at 2 min, whereupon this was maintained for 2 min and then decreased to 0% at 6 min. Finally, the last 4 min of analysis was kept at 90% B. The flow rate was 0.6 mL/min and the injection volume was 10 µL. A sample volume of 6 μL was injected into the analytical column for compound analysis.

Mass spectrometric analyses were accomplished on an Applied Biosystems MDS Sciex (Concord, ON, Canada) API 3200 triple quadrupole mass spectrometer equipped with an electrospray ionization (ESI) interface. ESI ionization was performed in the positive ionization mode. High purity nitrogen used as a sheath gas was generated with a nitrogen generator. All of the experiments were carried out in the positive-ion mode. The ion source parameters were as follows: ion spray voltage (IS): 5500 V; nebulizer gas (gas 1): 25 psi; turbo gas (gas 2): 20 psi; temperature of the heated nebulizer (TEM): 350 °C; curtain gas (CUR): 10 psi. The mass spectrometer was operated with a dwell time of 200 ms. To find the optimal parameters of the ion path and ion source of the studied compound, quantitative optimization was carried out through direct infusion of the standards using a Hamilton syringe pump (Hamilton, Reno, NV, USA). The multiple reaction monitoring (MRM) mode of the dominant product ion for each compound was realized using optimal conditions. The monitored ions with values *m*/*z* were: 329.0/284.0 for U-47700; 314.9/283.9 for *N*-desmethyl-U-47700; 301.0/172.9 for *N*,*N*-didesmethyl-U-47700; and 336.0/286.0 for U-47700-*D*_6_ (IS). The total analysis time for a single sample was 12 min. Retention times (RT) for U-47700 and its metabolites were as follows: 8.5 for U-47700 and U-47700-*D*_6_; 8.23 for *N*,*N*-didesmethyl-U-47700, and 8.3 for *N*-desmethyl-U-47700. Data acquisition and processing were performed using the Applied Biosystems Analyst version 1.4.2 software.

#### 2.3.3. Calibration Curve and Quantification

The concentrations of U-47700 and its metabolites (*N*-desmethyl-U-47700 and *N*,*N*-didesmethyl-U-47700) in the samples were calculated using the calibration curve that was prepared on the same day and analysed in the same analytical run. Calibration curves were constructed after analysing tissue samples collected from naive rats. The homogenates were spiked with U-47700 and its metabolites at the following concentration: blank, 0.1, 1, 10, 25, 50, 100 ng/mg. U-47700-*D*_6_ was used as the IS. These samples were analysed according to the procedure described for sample preparation (Section 2.3.1). Calculations: for U-47700 relative to IS (quantitative) and as a percentage for metabolites. Method validation data are presented in the Tables S1 and S2 included in the Supplementary Materials.

### 2.4. Statistical Analyses

All data were expressed as means ± S.E.M. In biochemical assays, statistical analyses were performed with a t-student test. *p* < 0.05 was considered statistically significant.

## 3. Results and Discussion

In many studies, regardless of the determined parameters (Ki/IC50/Kd/EC50), in vitro methods were used first. In affinity studies, the Ki values for U-47700 were Ki = 0.91; 110 and 480 nM for μ, κ, and δ receptors, respectively. For the comparison of Ki to morphine, it is 0.213; 27.9; 111 nM for μ, κ, and δ, respectively, whereas in the activity assays, it is 140; 201 and 4540 nM for μ, κ, and δ, respectively (comparison of EC50 to morphine: 31; 83; 870 nM). In vitro studies suggest that because U-47700 has a lower affinity for receptors than morphine, it should act less well. The pharmacokinetic profile of the com-pound plays a significant role in U-47700 for µi opioid receptors (in relation to morphine), resulting from other physicochemical parameters. U-47700 is much more lipophilic in relation to morphine [2,12,13,14]. Using U-47700 as an example, as well as its pharmacokinetic parameters and metabolic profile, we wanted to present a contemporary approach to the issue of testing the toxicity assessment of new psychoactive substances.

The problem of substance abuse that affects the central nervous system is still valid despite constantly revised legal regulations. This is due to the fact that ongoing basic re-search yields little knowledge, while experimentation with new psychoactive substances carries a high health risk, especially among young people. Adding to the risk is the fact that many NPS undergo biotransformation in the body to equally active (or often more potent) derivatives. In our work, we undertook a study of the biotransformation of a synthetic opioid—compound U-47700. In the case of this compound, its metabolites were found to have a lower toxic potential and a weaker affinity for opioid receptors [14].

In the first stage, an in silico test was performed with the use of ADMET Predictor software (version 10.4.0.5; Simulations Plus, Inc.; Lancaster, CA, USA), where proposals for 13 derivative compounds were obtained based on the physicochemical properties of the starting structure. The next step involved in vitro studies using HLM and commercially sourced S9 fractions. In order to complete the research, a simple in vivo experiment was performed on an animal model (rats), which made it possible to confirm certain assumptions and to track the distribution of the studied metabolites in tissues such as blood, brain and liver. The conducted research is a precursor to further analysis, expanded to include all possible metabolites formed in various types of tissue—from blood to nervous tissue. To date, studies on NSOs’ metabolism have been limited due to the variability of NPSs. In addition, where poisoning with this compound is concerned, the first priority is to save the poisoned person before then determining the cause of the poisoning. Confirmation of the presence of the parent substance is sufficient to initiate treatment. However, an equally important aspect is the issue of the metabolites formed, including their pharmacological activity, which contributes to many factors, including the course of poisoning. By analysing the scientific literature describing the metabolism of the opioid U-47700, one can notice the growing interest in the biotransformation of this compound. Undoubtedly, an important element of such research is the possibility of using currently available research tools, such as in silico analysis and in vitro tests. These are tools that allow you to conduct research in accordance with the 3Rs (replacement, reduction, refinement) rules and provide a lot of valuable information allowing for a more complete interpretation of the results from poisoning cases [15,16].

The analyzes performed with the ADMET Predictor software indicate that the major metabolic pathway for U-47700 involves the formation of desmethyl derivatives (M1 and M2) of the parent compound (Figure 1). The probability score for the formation of these metabolites was 94% (a probability score with 100% being the maximum likelihood of generation). The demethylation process of M1 leading to *N*-desmethyl-M1 and hydroxylation of M1 to hydroxy-M1 are residual processes, with the probability of the formation of metabolites at 1% and 5%, respectively. Similarly, the processes of hydroxylation of the U-47700 aromatic ring, leading to hydroxy-U-47700, and hydroxylation of the alkyl group, leading to hydroxy-U-47700′, are residual processes, with the probability of metabolites’ formation at 3%. One of the many features of the ADMET Predictor is the ability to predict biotransformation pathways of a given substance along with the determination of specific isoenzymes responsible for particular transformations (Table 1). Information on the expected quantitative content of metabolites is also available. The program also allows for the determination of various pharmacokinetic properties of the obtained metabolites. The in silico study was treated as a preliminary procedure, seeking information only on phase I metabolites, which should be expected in subsequent studies. By analysing the scheme obtained in the ADMET Predictor program, it can be concluded that the proposed metabolites of opioid U-47700 are consistent with the metabolites found in in vitro studies of this compound in the literature [17,18,19,20]. The M4 and M4/M3 metabolites (demethylated derivatives U-47700) were indicated as the most important compounds formed (Figure 1). In the case of both of these compounds, their formation would be mainly due to the isoenzymes CYP1A2 and 2C19, which were also indicated as the main isoenzymes responsible for the demethylation process and the formation of the main metabolites of U-47700 (Figure 1 and Table 1). The analysis of the in silico results reveals that CYP enzyme isoforms such as 3A4 (involved in the hydroxylation process), as well as 2C9 and 2D6, are involved in the biotransformation of U-47700.

In vivo studies using HLM fractions showed a rapid depletion of the parent substance—U-47700 for 15 min. At the same time, a build-up of M1 and M2 metabolites was observed, with M2 continuing its rapid growth for 90 min, and M1 declining slightly for 15 min to the 90 min time point (Figure 2A). In the case of the S9 fraction, a slower yet no less dynamic depletion of U-47700 was observed at 90 min. At the same time, a build-up of the M1 metabolite was observed until 30 min, followed by a sharp decline at 60 min and a renewed build-up up to 90 min. In the case of the M2 metabolite, a dynamic build-up up to 90 min was observed (Figure 2B). No major differences could be observed between incubations with human cytosolic fraction S9 or when utilizing human microsomal fractions. Apart from two metabolites, both methods rendered the same qualitative metabolic profile, with quantitative differences. As a result, both protocols applied in this study can be used to study in vitro human liver biotransformation reactions.

**Figure 3:**
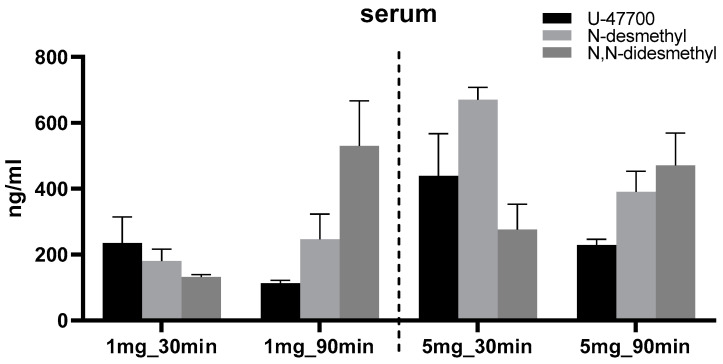

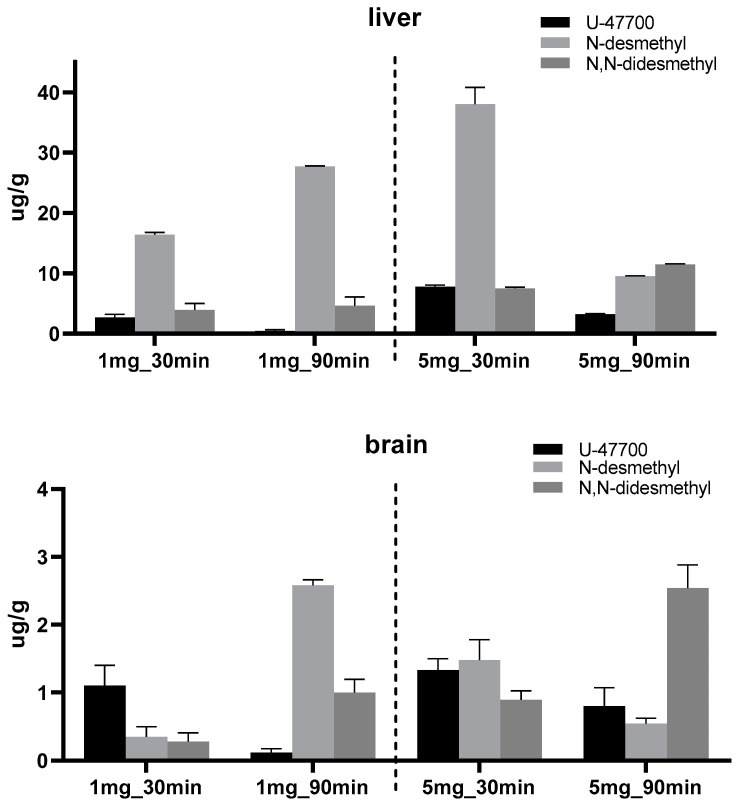
Content of U-47700 and major metabolites in tissues (*n* = 3/group; mean +/− SEM).

The results obtained from the experiment in the animal model show a difference in the biotransformation pathways of U-47700. When using a lower dose of the compound (1 mg/kg b.w.) in animals, the formation of the *N*-desmethyl derivative U-47700 was mainly observed in the examined tissues (liver and brain) after 90 min. The profile of the formed metabolites was similar to that in the case of the higher dose—5 mg/kg b.w. as shown in Figure 3 and Table 2—only in the serum. The higher dose of U-47700 resulted in the appearance of large amounts of *N*-desmethyl-U-47700 after both 30 min and 90 min. In animals dosed with 5 mg/kg b.w., U-47700 appears to be biotransformed more slowly, especially in the liver, but the *N*,*N*-didesmethyl derivative pathway predominates. A *p.o.* dose of 5 mg/kg b.w., in our opinion, could be considered subliminally toxic to rats, as the development of cataplexy symptoms was observed until the 90 min time point. Exceeding this dose could result in death through a mechanism of respiratory depression before the end of the experiment. Nordmeier et al. (2021) have shown that the major metabolites of U-47700 have significantly lower biological potential than the parent compound. Therefore, it can be assumed that the pathway involving the formation of *N*,*N*-didesmethyl-U-47700 leads to a reduction in the toxicity of the compound [19].

Due to the absence of controlled clinical studies of U-47700, in vitro and in vivo (animal models) studies can be used to fill an important void in the available data, which can be used for clinical and forensic purposes. The obtained metabolic profile of U-47700 in the tested in vitro and in vivo models is basically consistent with the metabolic profile in terms of quality (M1–M4 metabolites) obtained in the studies of biological material (serum, blood, urine) from patients hospitalized due to poisoning or autopsy material (blood, urine) from others who have died from an opioid overdose [11,21].

Quantitative studies of the metabolic profile of U-47700 in the animal model for doses of 1 and 5 mg/kg b.w., for the two time points of 30 and 90 min, indicated that the concentrations of U-47700 and its metabolites M1 and M2 were in the ranges of 113–259 ng/mL; 179–625 ng/mL and 132–530 ng/mL, respectively (Table 2). These ranges are included in those reported with most cases involving poly-drug use, which are for U-47000 and its metabolites M1 and M2 in the range 1.1–24,000 ng/mL, 2.0–7520 and 0.7–1947 ng/mL, respectively [9,20]. Quantitative data on metabolites in other tissues (liver or brain) are not available. The obtained concentration ranges of U-47700 in the animals study overlap with the blood concentrations measured in non-fatal U-47700 intoxications in humans (7.6–394 ng/mL in serum samples and 94–282 ng/mL in blood samples), suggesting that the rat model has a translational value. A few cases have reported U-47700 as the sole substance involved in both non-fatal intoxications and fatal drug overdoses. Even in these instances, where the concentrations of U-47700 overlap for non-fatal and fatal cases, it is impossible to specifically identify a lethal concentration of the drug [21,22,23].

The in vitro and in vivo study of the metabolic profile of NPS is a useful tool for providing data. This may be useful in terms of diagnosis, assessment of the severity of poisoning and treatment for the purposes of clinical toxicology, or the toxicological distinction of “rapid deaths” and “delayed deaths” [24,25], predicting possible post mortem redistribution processes and their impact on the final result of determinations for the purposes of medical and forensic opinion.

## Figures and Tables

**Table 1:** List of the main metabolites of U-47700 along with the proposed enzymes participating in the biotransformation according to ADMET Predictor software.

Symbol	Name	Chemical Structure	Proposed Enzymes Involved in Biotransformation
	U-47700	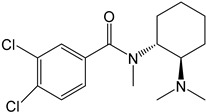	
M1	*N*-desmethyl- U-47700	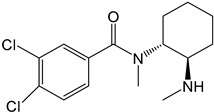	CYP 2C19 CYP 1A2 CYP 2C9 CYP 3A4 CYP 2D6
M2	*N*,*N*-didesmethyl- U-47700	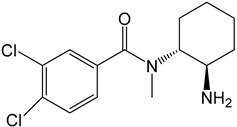	CYP 2C19 CYP 1A2 CYP 2C9 CYP 3A4 CYP 2D6
M3	hydroxy-*N*-desmethyl- U-47700	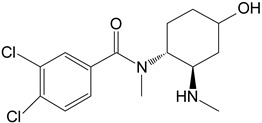	CYP 3A4 CYP 1A2 CYP 2C9
M4	hydroxy-*N*,*N*-didesmethyl- U-47700	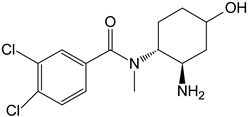	CYP 3A4 CYP 2C9
desmethyl-M1		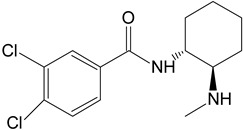	
hydroxy-M1		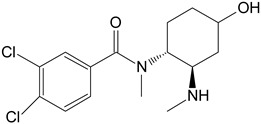	CYP 2C9 CYP3A4
hydroxy-U47700		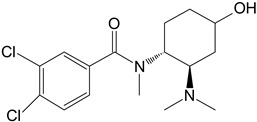	CYP 3A4
keto- U47700		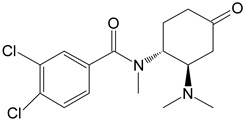	CYP 2C9 CYP 3A4
hydroxy-U47700′		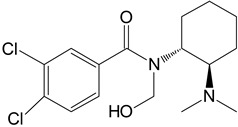	CYP3A4
keto- U47700′		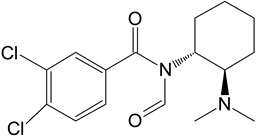	CYP 1A2 CYP 3A4
M3′		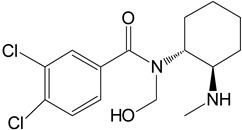	CYP 1A2 CYP 3A4 CYP 2C9 CYP 2D6
M1′		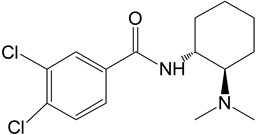	
hydroxy- M1′		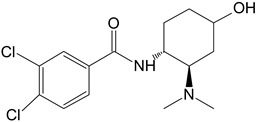	CYP 3A4 CYP 2C9
desmethyl- M1′		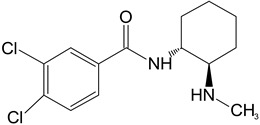	CYP 2C19 CYP 1A2 CYP 2C9 CYP2D6 CYP3A4

**Table 2:** The concentrations of the tested compounds in the biological material obtained from the in vivo experiment (*n* = 3; mean +/− SEM).

Dose (mg)	Tissue (ng/mL) +/− SEM or (µg/g) +/− SEM	Time (min)	U-47700	*N*-Desmethyl- U-47700	*N*,*N*-Didesmethyl-U-47700
1 mg	Serum	30′	235 +/− 79.0	180 +/− 36.6	132 +/− 7.34
		90′	113 +/− 8.4	246 +/− 77.2	530 +/− 137
5 mg		30′	439 +/− 128	670 +/− 37.5	276 +/− 77.1
		90′	229 +/− 17	390 +/− 63.2	471 +/− 98
1 mg	Liver	30′	2.68 +/− 0.510	1.64 +/− 0.381	3.92 +/− 1.070
		90′	0.45 +/− 0.210	0.947 +/− 0.095	4.62 +/− 1.490
5 mg		30′	7.79 +/− 0.240	3.81 +/− 2.770	0.75 +/− 0.196
		90′	3.26 +/− 0.066	1.08 +/− 0.075	1.14 +/− 0.136
1 mg	Brain	30′	1.1 +/− 0.300	0.353 +/− 0.145	0.28 +/− 0.129
		90′	0.12 +/− 0.054	2.58 +/− 0.080	0.997 +/− 0.197
5 mg		30′	1.33 +/− 0.170	1.48 +/− 0.299	0.891 +/− 0.134
		90′	0.8 +/− 0.270	0.545 +/− 0.080	2.54 +/− 0.341

## Data Availability

The original contributions generated for this study are included in the article; further inquiries can be directed to the corresponding author.

## Supplementary Materials

The following are available online at https://www.mdpi.com/article/10.3390/toxics11030220/s1, Table S1: Analytical parameters of the calibration equations for the determination of U-47700 and its major metabolites in biological tissues—liver, serum and brain (n = 5); Table S2: Accuracy and precision for U-47700 and its major metabolites in biological tissues—liver, serum and brain samples.
