# Supplementary material for: Metabolic Evaluation of Synthetic Opioids on the Example of U-47700 with the Use of In Vitro and In Vivo Methods for Forensic Toxicology Application
Source: Toxics. 2023 Feb 25;11(3):220. doi: 10.3390/toxics11030220 (PMC10053220; doi:10.3390/toxics11030220)
Supplement: Supplementary file 1 [file toxics-11-00220-s001.zip › toxics-2222894-supplementary.pdf]

Table

Analytical parameters of the calibration equations for the determination of U-47700 and its major metabolites in biological tissues – liver, serum and brain (n = 5).

	Tissue	Concentration range (ng/100 mg)	Regression equation	Correlation coefficient R^2
U-47700	Serum	25 - 5000	y=0.0018x-0.0334	0.9987
	Liver		y=0.0017x-0.0442	0.9982
	Brain		y=0.002x+0.034	0.9994
N-desmethyl-U-47700	Serum	25 - 5000	y=0.0012x+0.0071	0.9979
	Liver		y=0.0009x+0.1056	0.9761
	Brain		y=0.0013x+0.0454	0.9957
N,N-didesmethyl-U-47700	Serum	25 - 5000	y=0.031x-1.1227	0.9863
	Liver		y=0.0293x-1.19	0.9797
	Brain		y=0.0245x+0.3201	0.998

Accuracy and precision for U-47700 and its major metabolites in biological tissues – liver, serum and brain samples.

U-47700		Concentration added	%R.S.D.
Intraday (n = 4)	Serum (ng/100ml)	25	1.2
		250	2.2
		5000	7.72
	Liver (ng/100mg)	100	6.33
		500	7.64
		5000	4.88
	Brain (ng/100mg)	25	11.80
		250	1.23
		5000	10.28
N-desmethyl-U-47700			
Intraday (n = 4)	Serum (ng/100ml)	25	14.31
		250	3.47
		5000	11.19
	Liver (ng/100mg)	100	8.98
		500	8.24
		5000	---
	Brain (ng/100mg)	25	2.69
		250	10.02
		5000	---
N,N-didesmethyl-U-47700			
Intraday (n = 4)	Serum (ng/100ml)	25	14.19
		250	3.14
		5000	---
	Liver (ng/100mg)	100	13.44
		500	9.40
		5000	----
	Brain (ng/100mg)	25	9.69
		250	3.13
		5000	----
